# Immune Infiltration Analysis with the CIBERSORT Method in Lung Cancer

**DOI:** 10.1155/2022/3186427

**Published:** 2022-03-18

**Authors:** Meng Guan, Yan Jiao, Lili Zhou

**Affiliations:** ^1^Cancer Center, The First Hospital of Jilin University, Changchun 130031, China; ^2^Department of Hepatobiliary and Pancreatic Surgery, The First Hospital of Jilin University, Changchun 130031, China; ^3^Department of Radiology, The First Hospital of Jilin University, Changchun 130031, China

## Abstract

**Background:**

Immune infiltration of lung cancer (LC) is tightly related to clinical results. Nevertheless, past researches have not elucidated the diversities of functionally different cellular types making up the immunoresponse.

**Methods:**

In the present research, on the foundation of a deconvolution algorithm (CIBERSORT) and clinically annotated expression profiles, our team studied the tumor-infiltrating immune cells (TIICs) presenting in 502 LC samples and 49 normal samples in a comprehensive way. The fraction of 22 immunocyte subgroups was assessed to identify the relationship among every cellular type and survival and reaction to chemical therapies.

**Results:**

Consequently, profiles of immunity infiltration change remarkably between paired tumor and precancerous tissues, and the change can describe the diversity of individuals. Of the cellular subgroups studied, cancers without dendritic resting cells or with a decreased quantity of follicular helper T (Tfh) cells were related to the poor prognosis. Correlation analysis between different stages of LC and 22 immune cell subpopulations revealed that the amount of 14 immune cells in LC was remarkably related to tumor stage. The high expression of resting dendritic cells and follicular helper T cells predicted better prognostic value, and univariate analyses proved that two TIICs were significantly associated with patients' prognosis.

**Conclusions:**

To sum up, the data herein reveal that there may be subtle differences in the cell constituents of the immune infiltrate in LC, and those diversities may be vital determinating factors of prognostic results and reactions to therapies.

## 1. Introduction

Tumor-infiltrating immune cells (TIICs) are remarkably associated with prognostic results and determination of immune therapy targets in lung cancer (LC) [1]. LC is the major cause of tumor-related death across the globe and causes 1.6 million deaths each year [2]. Metastasis is responsible for the high death rate in LC While significant progress in treatment choices for LC sufferers was developed, patients suffered from LC have worse prognosis and limited treatment options [3, 4]. Moreover, LC is often diagnosed with terminal stage cancer, which makes only palliative treatments acceptable. The genome variations in carcinoma are deeply studied to distinguish sufferer subtypes with diverse prognostic results [5]. Accumulating evidence proved that the abnormal phenotypes of carcinoma are determined by the sophisticated mutual effects of a variety of cellular types in the TME, especially TIICs [6, 7].

As an immune sensitive cancer, LC infiltrated by an inhomogeneous immune cell subpopulation of TIICs, such as T cells, DCs, macrophagus, neutrophils, mast cells, and the type, density, and site of TIICs in LC contains significant prognostic value [8]. Previous studies mainly relied on immunohistochemistry and flow cytometry to assess the profile of TIICs subtypes. However, flow cytometry requires precise and careful processing of samples, and immunophenotyping cannot identify enough immune populations [9, 10]. After Newman developed a bioinformatic tool-CIBERSORT algorithm, offer a speculation of the quantity of member cell types in a blended cellular population with genetic expression data [11, 12]. The CIBERSORT has the advantage in accurately evaluating the defined fraction of the 22 closely related type immune cells by only applying signature gens of bulk tumor samples. CIBERSORT enables immunocyte profiling by deconvolution of genetic expression micro array data [13]. In the present research, our team applied deconvolution algorithm (CIBERSORT) to assess the relative proportions of immunocytes in 49 adjacent samples and 502 LC samples.

In this research, we made use of CIBERSORT to evaluate the 22 TIIC subtypes in LC to elucidate TIICs' dedicated association with molecule subgroup, survival ratio, and reaction to chemical therapies. This study explains the association between the inhomogeneity of TIICs and illness development in LC.

## 2. Materials and Methods

### 2.1. Data Acquisition

We acquired genetic expression profiles of LC (*n* = 502) and healthy specimens (*n* = 49) and clinic features including medication history, histologic grade, pathologic stage, and survival information for patients with LC from the relevant sufferers from TCGA. In the case of whose prognosis data were not correlated with their expression profiles, our team went through the supplements of these missing information. RNA sequence data were standardized via the average-variance model at the observation level approach, which transformed enumeration data to result more like those from microarrays, as clinic data, excluding LC sufferers who had missing information of age, gender, TNM stage, local invasion, survival time, and disease-free survival. Then, LC patients (*n* = 463) with complete information were included. We manually arranged every expression information and corresponding clinical data. Our study followed the instruction of profiling TIICs with CIBERSORT [9].

### 2.2. Analyzation of TIICs

CIBERSORT analysis tool is a gene expression-based arithmetic, which uses a series of bar code genetic expression results (a “signature matrix” of 547 genes) to assess data of immunocyte constituents from bulk cancer specimens [14]. To realize the precise quantification of the fraction of 22 immunocyte types in LC samples, standardized genetic expression data sets were employed and sent to the CIBERSORT web portal (http://cibersort.stanford.edu/) and set the quantity of permutations to 1,000. An overall of 22 TIIC types and CIBERSORT metrics, such as CIBERSORT *p* value, Pearson's correlation coefficient, and root mean squared error (RMSE), was subjected to quantification for every specimen [15]. The statistic significance of the deconvolution results of the entire cellular subgroups was represented by the CIBERSORT *p* value, which was employed to exclude the deconvolution with less remarkable fit precision. For the purpose of meeting the demand of a CIBERSORT *p* ≤ 0.05, healthy specimens (*n* = 49) and LC specimens (*n* = 520) were chosen. Every sample was quantified under 22 types of TIICs and CIBERSORT metrics as Pearson correlation coefficient, CIBERSORT *p* value, and RMSE. CIBERSORT *p* value offers an identification of confidence in the outcomes. As *p* < 0.05, the evaluation of the inferred portion of immunocyte subsets evaluated by CIBERSORT was regarded reliable.

### 2.3. Statistics

Our team completed the statistic assays by *R* software 3.5.2 and IBM SPSS Statistics 20.0. The ideal prognostic model was analyzed by the LASSO Cox regression to evaluate immune cell subtypes using the glmnet package in *R* [16]. The layer clustering of immunocyte fractions was applied to reveal different immunocyte infiltrations among diverse specimens. We valued the levels of 22 TIIC subpopulations between 0 and 1 in this assessment. Applied *R* packages “Corrplot,” “Pheatmap,” and “Vioplot” determinate variations in the mixture of TIICs among these groups. Wilcoxon test was employed to assess the association among cancer grades and molecule-level subgroups of cancer and TIICs. Log rank test and Kaplan–Meier (K–M) curve was also applied to confirm the relationship between TIICs and survival. Multivariate analysis was employed for in-depth study to select independent predicting factors. AUC and cut-off value were acquired from ROC curve. “Limma” package was applied to analyze the differentially expressed gene (DEG), and filters were set at ∣log_2_FC | >1.3219 and FDR < 0.05. We verified that variation between inferred levels of TIIC cellular subset and survival was examined via Cox regressive method. Exerted assays for patients with/without LC to elucidate the basic difference in LC and for known violations of the Cox proportion risk hypothesis in TIIC levels may be an inherent characteristic that can characterize the diversities between individuals. Finally, the proportions of immune cells from 463 LC patients' tissues and 49 adjacent samples displayed distinct group-bias clustering and individual differences.

## 3. Results

### 3.1. Composition Difference of Immune Cells in LC Samples and Adjacent Samples

**Figure 1:**
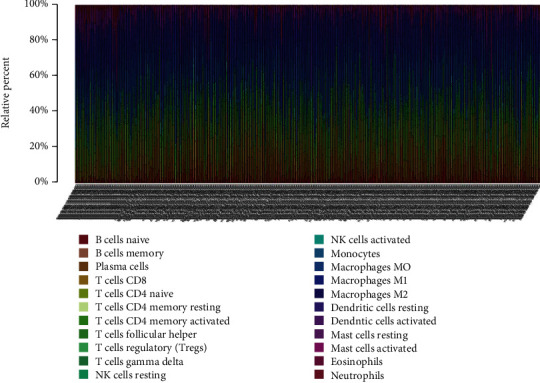
Histogram of the percentage of 22 immunocyte subgroups in LC.

**Figure 2:**
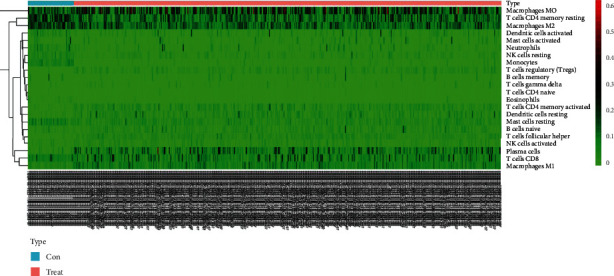
Heat map of the content of 22 immunocyte subgroups in lung cancer.

After the operation of manual selection, we enrolled 463 tumor tissues and 49 adjacent tissues as the training and validation cohorts, respectively, initially, normalizing the gene expression data with “Limma” package, followed by assessment of the difference of immune infiltration of LC specimens in 22 subtypes of immunocytes with the CIBERSORT algorithm and define the sum of 22 subsets immunocytes in every specimen as 1.  Figure 1 depicts the fraction of the entire 22 subtypes of immunocytes in each sample and as the hierarchical clustering revealed TIICs, such as NK cell resting, monocytes, and plasma cells displayed distinct distribution differences in LC samples and adjacent samples (Figure 2).

### 3.2. Correlation Degree of 22 Immune Cell Subgroups in Each Sample

**Figure 3:**
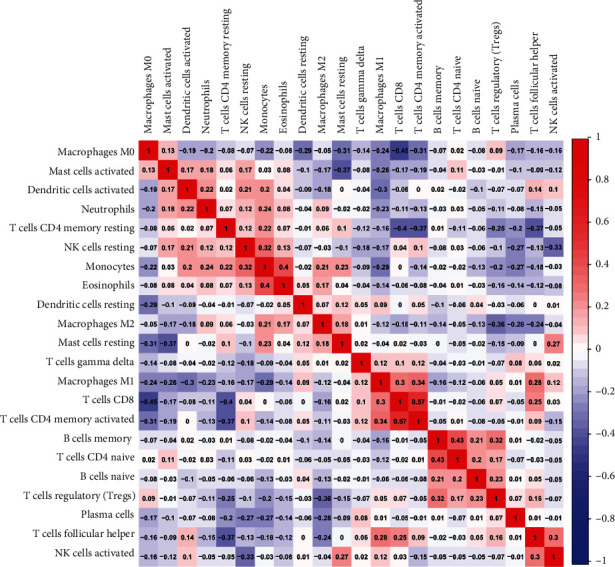
Correlation degree of 22 immunocyte subgroups in every specimen.

Notably, it was the fractions of immunocytes that changed remarkably in LC specimens and adjacent samples. We could easily find that T cell CD 4 memory activated and T cell CD8 exerted a remarkable positive association; nevertheless, an obvious negative association between T cell CD8 and macrophage M0 was showed by average linkage clustering (Figure 3).

### 3.3. Expression Levels of 22 Immunocyte Subsets in LC and Normal Groups

**Figure 4:**
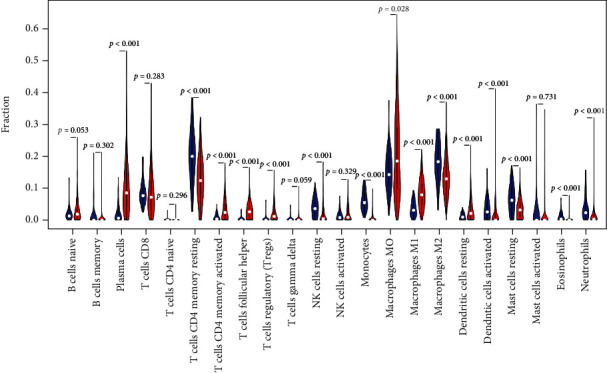
Violin chart of the expression levels of 22 immunocyte subgroups in LC and normal groups.

The violin plot indicated that obvious differences existed in the distribution of 14 out of 22 immunocytes, like T cell CD 4 memory resting (*p* < 0.001), plasma cells (*p* < 0.001), T cell CD 4 memory activated (*p* < 0.01), and between LC samples and adjacent samples cohorts (Figure 4). To sum up, all above results demonstrated that the inhomogeneity of TIICs in lung cancer is undoubtedly and of which might employ a crucial factor in the malignant development of LC.

### 3.4. Immunocyte Comparison Responding to the Prognostic Results of LC

**Figure 5:**
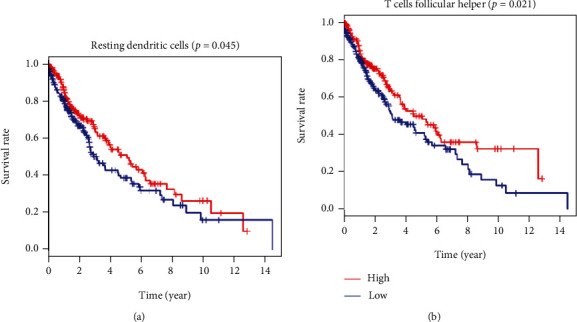
TIICs remarkably related to the prognostic results of sufferers with lung cancer. (a, b) K-M curves displayed high expression level of immunocytes, such as resting dendritic cells (*p* = 0.045), T cell follicular helper cells (*p* = 0.021), and associated with good OS.

The clinical data from TCGA databases was acquired and then eliminated the samples with less than 30 days' survival time and manually organized the expression profiles of every specimen and relevant clinic data. Total sample was randomly separated into the experiment group and validation group, and the ratio is 7 : 3 (experiment group: validation group). Univariate analyses were used to value immune cell infiltration and corresponding survival time.  Table 1 and Figure S1 show the survival analysis results of 22 immune cell subpopulations.  Figure 5 shows the high expression of dendritic cells at rest (*p* = 0.045) and T cell follicular helper cells (*p* = 0.021). It has a good predictive value for the prognosis. The univariate analysis proves that the two TIICs are significantly related to the patient's prognosis and are of great significance for postoperative immunotherapy of lung cancer.

## 4. Discussion

In this study, we report an extensive evaluation of LC TIICs in 502 tumor samples and 49 adjacent tissues. The CIBERSORT analytical tool gives us a great advantage to specifically analyze the essential fractions of 22 subpopulations TIICs in bulk cancer specimens. And the insight of TIICs may be helpful to explain the initiation and development of LC. Moreover, genes which are uniquely expressed in LC samples could be precious predictor in diagnosis and prognosis, but little research has highlighted the differential distribution of immunocytes between diverse constituents.

The complex and unique communities of cell life are called microenvironments by scientists. The microenvironment has many characteristics that affect cell growth, behavior, and how to communicate with other cells nearby [17–19]. Different types of tumor cells interact with different types of immune cells. These immune cells have the function of helping or attacking tumors [20, 21]. The hierarchical clustering revealed that TIICs, such as NK cell resting, monocytes, and plasma cells, displayed distinct distribution differences in LC samples and adjacent samples. The violin plot indicated that an obvious difference existed in the distribution of 14 out of 22 immunocytes, like T cell CD4 memory resting (*p* < 0.001), plasma cells (*p* < 0.001), T cell CD4 memory activated (*p* < 0.01), and between LC samples and adjacent samples cohorts.

And we could easily find that T cell CD4 memory activated and T cell CD8 exerted a remarkable positive association; nevertheless, an obvious negative association between T cell CD8 and macrophage M0 was showed by average linkage clustering. CD4+ T cells serve as a vital immunocyte in the immunosystem of mankind. CD4 is primarily expressed in Th cells, which can realize the binding to the nonmultipeptide areas of MHC class II molecules and participate in the recognition of antigens by T cell antigen receptors (TCR) [22, 23]. Signal transduction was as follows: relevant research has discovered that in tumor immunity, CD4+ T cells can activate CD8+ T cells through a variety of mechanisms to differentiate into cytotoxic T lymphocytes (CTL), while maintaining and strengthening the antitumor response of CTL [24]. In recent years, studies have found that macrophages account for 50% of the total weight of tumors. These cander-related macrophagus not only stop T cells from eliminating oncocytes but excrete growth factors to facilitate oncocytes and cancer angiogenic activities, causing the spread of cancer cells [25–28].

Univariate analyses were used to value immune cell infiltration and corresponding survival time. Highly expression of resting dendritic cells (*p* = 0.045) and follicular helper T (Tfh) cells (*p* = 0.021) predicted a better prognostic value, and univariate analyses prove that two TIICs were significantly associated with patients' prognosis. Dendritic cell (DC) is an important antigen-presenting cell (APC), which can strongly stimulate resting T cells [29, 30]. It is the main APC that activates naive T cells in the body, and Tfh cells are a new CD4+ helper T cell subgroup. More and more studies have shown that Tfh cells and their cell factors are vital for tumors and autoimmune diseases [31–33].

In conclusion, our study revealed distinct immune phenotypes for molecular LC subclasses. Hence, our team suggests that differences in TIIC fractions may be an inherent characteristic that can characterize the difference of individuals. Those discoveries strengthen the comprehension of immunoresponses in LC cancers and might exert an indispensable effect on the design of effective immunotherapeutic strategies.

## Figures and Tables

**Table 1:** Survival analysis results of 22 immune cell subsets.

Gene	*p* value
B cell naive	0.218408
B cell memory	0.961728
Plasma cells	0.738127
T cell CD8	0.962302
T cell CD4 naive	0.220519
T cell CD4 memory resting	0.138559
T cell CD4 memory activated	0.796954
T cell follicular helper	0.021364
T cell regulatory (Tregs)	0.801911
T cell gamma delta	0.215271
NK cell resting	0.45362
NK cell activated	0.257787
Monocytes	0.090153
Macrophage M0	0.934702
Macrophage M1	0.5506
Macrophage M2	0.629833
Dendritic cell resting	0.044586
Dendritic cell activated	0.313067
Mast cell resting	0.564689
Mast cell activated	0.403959
Eosinophils	0.285823
Neutrophils	0.373427

## Data Availability

The original data used to support the findings of this study are available from the corresponding author upon request.

## Supplementary Materials

Supplementary Materials Figure S1: Kaplan-Meier curves of 22 immune cell subsets in lung cancer.

## References

[1] Meza R., Meernik C., Jeon J., Cote M. L. (2015). Lung cancer incidence trends by gender, race and histology in the United States, 1973–2010. *PLoS One*.

[2] Thorsson V., Gibbs D. L., Brown S. D. (2018). The immune landscape of cancer. *Immunity*.

[3] Woodard G. A., Jones K. D., Jablons D. M. (2016). Lung cancer staging and prognosis. *Cancer Treatment and Research*.

[4] Hua X., Chen J., Wu Y., Sha J., Han S., Zhu X. (2019). Prognostic role of the advanced lung cancer inflammation index in cancer patients: a meta-analysis. *World Journal of Surgical Oncology*.

[5] Bar J., Ofek E., Barshack I. (2019). Transformation to small cell lung cancer as a mechanism of resistance to immunotherapy in non-small cell lung cancer. *Lung Cancer*.

[6] Zhu G., Pei L., Yin H. (2019). Profiles of tumor-infiltrating immune cells in renal cell carcinoma and their clinical implications. *Oncology Letters*.

[7] Gnjatic S., Bronte V., Brunet L. R. (2017). Identifying baseline immune-related biomarkers to predict clinical outcome of immunotherapy. *Journal for Immunotherapy of Cancer*.

[8] Gentles A. J., Newman A. M., Liu C. L. (2015). The prognostic landscape of genes and infiltrating immune cells across human cancers. *Nature Medicine*.

[9] Chen B., Khodadoust M. S., Liu C. L., Newman A. M., Alizadeh A. A. (2018). Profiling tumor infiltrating immune cells with CIBERSORT. *Methods in Molecular Biology*.

[10] Lu J., Li H., Chen Z. (2019). Identification of 3 subpopulations of tumor-infiltrating immune cells for malignant transformation of low-grade glioma. *Cancer Cell International*.

[11] Bremnes R. M., Busund L. T., Kilvaer T. L. (2016). The role of tumor-infiltrating lymphocytes in development, progression, and prognosis of non-small cell lung cancer. *Journal of Thoracic Oncology*.

[12] Bremnes R. M., Al-Shibli K., Donnem T. (2011). The role of tumor-infiltrating immune cells and chronic inflammation at the tumor site on cancer development, progression, and prognosis: emphasis on Non-small cell lung cancer. *Journal of Thoracic Oncology*.

[13] Rohr-Udilova N., Klinglmuller F., Schulte-Hermann R. (2018). Deviations of the immune cell landscape between healthy liver and hepatocellular carcinoma. *Scientific Reports*.

[14] Newman A. M., Liu C. L., Green M. R. (2015). Robust enumeration of cell subsets from tissue expression profiles. *Nature Methods*.

[15] Chen F., Yang Y., Zhao Y., Pei L., Yan H. (2019). Immune infiltration profiling in nonsmall cell lung cancer and their clinical significance: study based on gene expression measurements. *DNA and Cell Biology*.

[16] Narayanan S., Kawaguchi T., Peng X. (2019). Tumor infiltrating lymphocytes and macrophages improve survival in microsatellite unstable colorectal cancer. *Scientific Reports*.

[17] Dranoff G. (2014). Cancer immunology research: a one-year anniversary. *Cancer Immunology Research*.

[18] Miller J. F., Sadelain M. (2015). The journey from discoveries in fundamental immunology to cancer immunotherapy. *Cancer Cell*.

[19] Roth P., Eisele G., Weller M. (2012). Immunology of brain tumors. *Handbook of Clinical Neurology*.

[20] Dumauthioz N., Labiano S., Romero P. (2018). Tumor resident memory T cells: new players in immune surveillance and therapy. *Frontiers in Immunology*.

[21] Walsh S. R., Simovic B., Chen L. (2019). Endogenous T cells prevent tumor immune escape following adoptive T cell therapy. *The Journal of Clinical Investigation*.

[22] Taniuchi I. (2018). CD4 helper and CD8 cytotoxic T cell differentiation. *Annual Review of Immunology*.

[23] Zhang C., Ding H., Huang H. (2019). TCR repertoire intratumor heterogeneity of CD4+ and CD8+ T cells in centers and margins of localized lung adenocarcinomas. *International Journal of Cancer*.

[24] Cullen J. G., Mcquilten H. A., Quinn K. M. (2019). CD4+T help promotes influenza virus-specific CD8+T cell memory by limiting metabolic dysfunction. *Proceedings of the National Academy of Sciences of the United States of America*.

[25] Cassetta L., Pollard J. W. (2018). Targeting macrophages: therapeutic approaches in cancer. *Nature Reviews. Drug Discovery*.

[26] Nielsen S. R., Schmid M. C. (2017). Macrophages as key drivers of cancer progression and metastasis. *Mediators of Inflammation*.

[27] Ostuni R., Kratochvill F., Murray P. J., Natoli G. (2015). Macrophages and cancer: from mechanisms to therapeutic implications. *Trends in Immunology*.

[28] Salmi M. (2017). Macrophages and cancer. *Duodecim*.

[29] Macri C., Pang E. S., Patton T., O’Keeffe M. (2018). Dendritic cell subsets. *Seminars in Cell & Developmental Biology*.

[30] Waisman A., Lukas D., Clausen B. E., Yogev N. (2017). Dendritic cells as gatekeepers of tolerance. *Seminars in Immunopathology*.

[31] Gu-Trantien C., Migliori E., Buisseret L. (2017). CXCL13-producing TFH cells link immune suppression and adaptive memory in human breast cancer. *JCI Insight*.

[32] Poultsidi A., Dimopoulos Y., He T. F. (2018). Lymph node cellular dynamics in cancer and HIV: what can we learn for the follicular CD4 (Tfh) Cells?. *Frontiers in Immunology*.

[33] Vinuesa C. G., Linterman M. A., Yu D., MacLennan I. C. M. (2016). Follicular helper T cells. *Annual Review of Immunology*.

