# Supplementary figures and images for: Immune Infiltration Analysis with the CIBERSORT Method in Lung Cancer
Source: Dis Markers. 2022 Mar 18;2022:3186427. doi: 10.1155/2022/3186427 (PMC8956442; doi:10.1155/2022/3186427)

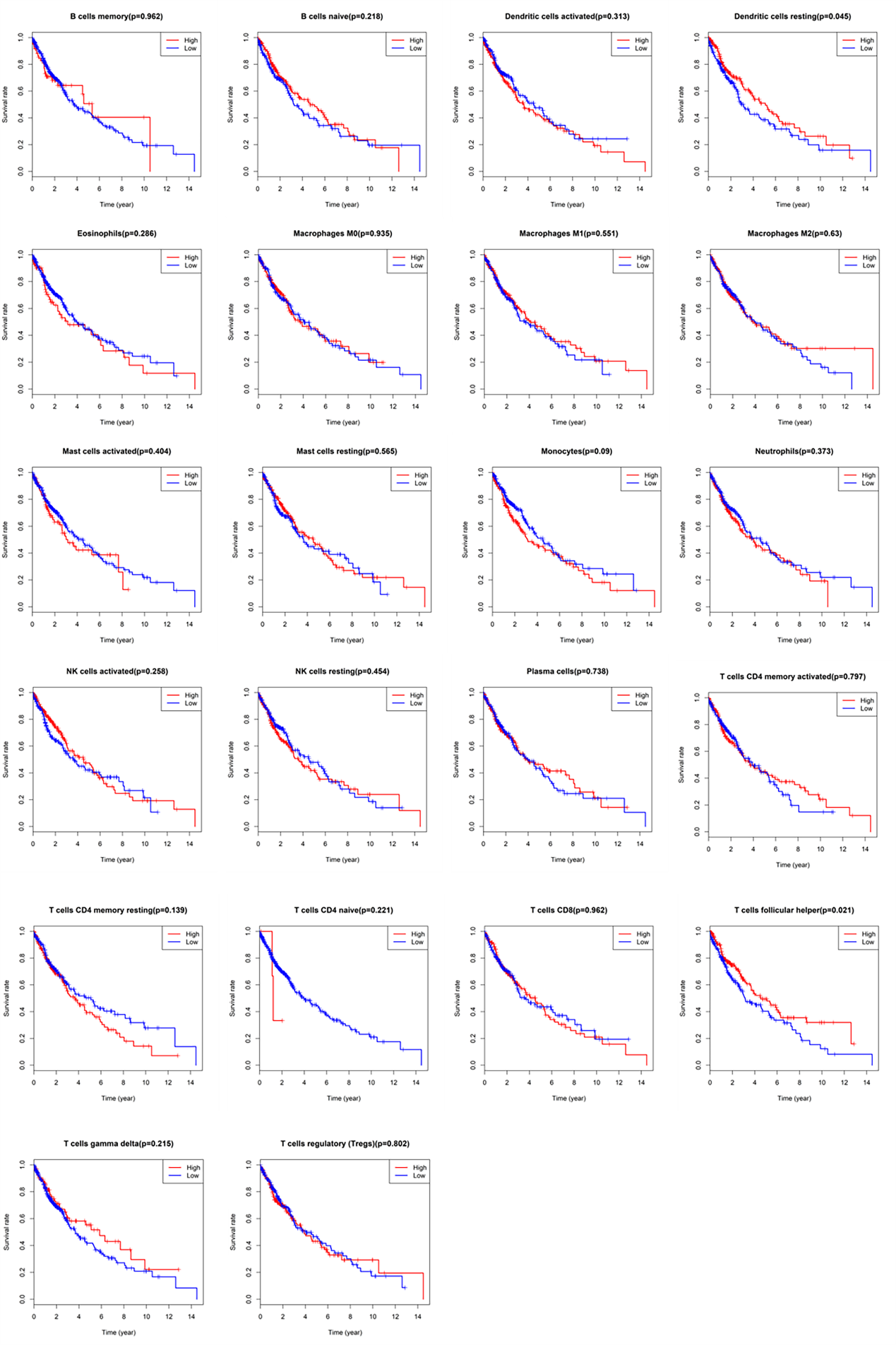


**Supplementary figure 1.** Kaplan-Meier curves of 22 immune cell subsets in lung cancer.

Supplement: Supplementary Materials — Figure S1: Kaplan-Meier curves of 22 immune cell subsets in lung cancer. [file 3186427.f1.DOCX]
